# Cardiogenic and Myogenic Gene Expression in Mesenchymal Stem Cells After 5-Azacytidine Treatment

**DOI:** 10.4274/Tjh.2012.0161

**Published:** 2013-06-05

**Authors:** Aungkura Supokawej, Pakpoom Kheolamai, Kuneerat Nartprayut, Yaowalak U-pratya, Sirikul Manochantr, Methichit Chayosumrit, Surapol Issaragrisil

**Affiliations:** 1 Mahidol University, Faculty of Medical Technology, Department of Clinical Microscopy, Bangkok, Thailand; 2 Thammasat University, Faculty of Medicine, Division of Cell Biology, Department of Pre-clinical Science, Pathumthani, Thailand; 3 Mahidol University, Faculty of Medicine, Division of Hematology, Department of Medicine, Siriraj Hospital, Bangkok, Thailand; 4 Mahidol University, Faculty of Medicine, Siriraj Hospital, Siriraj Center of Excellence for Stem Cell Research, Bangkok, Thailand

**Keywords:** mesenchymal stem cells, differentiation, Cardiomyocyte, Myocyte

## Abstract

**Objective:** 5-Azacytidine is a hypomethylating agent that is used for the treatment of myelodysplastic syndrome. This histone modifier is widely employed and plays a nonspecific role in influencing the differentiation capability of stem cells. The ability of bone marrow mesenchymal stem cells to differentiate into cardiomyocyte- and myocyte-like cells after exposure to 3 different doses of 5-azacytidine has been evaluated and compared. The aim of the study was to optimize the effective dose of 5-azacytidine for promoting the cardiomyocyte and myocyte differentiation capabilities of human mesenchymal stem cells (MSCs).

**Materials and Methods:** Human bone marrow aspirations were collected from healthy donors. MSCs were used for the study of mesodermal differentiation. MSCs were cultured to promote osteoblast differentiation and adipocyte differentiation. The evaluation of osteogenic or adipogenic properties was then performed through immunocytochemical staining. BMMSCs were trypsinized into single-cell suspensions and then prepared for flow cytometric analysis. The MSCs were treated with 5, 10, or 15 μM 5-azacytidine for 24 h and then cultured for 3 weeks. Total RNA was extracted from untreated and 5-azacytidine–treated cells. Troponin T and GATA4 antibodies were used as cardiogenic markers, whereas myogenin and MyoD antibodies were used as myocyte markers.

**Results:** The morphology and growth rate of MSCs that were treated with any of the 3 doses of 5-azacytidine were similar to the morphology and growth rate of control MSCs. An immunofluorescence analysis examining the expression of the cardiac-specific markers GATA4 and troponin T and the skeletal muscle-specific markers MyoD and myogenin revealed that cells treated with 15 μM 5-azacytidine were strongly positive for these markers. Real-time RT-PCR results were examined; these amplifications indicated that there were higher expression levels of cardiac- and skeletal muscle-specific mRNAs in MSCs treated with 15 μm 5-azacytidine than in MSCs that had either been treated with lower doses of 5-azacytidine or left untreated.

**Conclusion:** MSCs treated with 5-azacytidine demonstrated the capacity to differentiate into both cardiomyocytes and skeletal myocytes, and 15 μM 5-azacytidine could be the optimal dose of this drug. Other promoting factors should be examined to investigate the possibility of promoting the differentiation of MSCs into specific cell types.

**Conflict of interest:**None declared.

## Abstract (tr)

**Amaç:** 5-Azasitidin miyelodisplastik sendrom tedavisinde kullanılan hipometile edici bir ajandır. Bu histon değiştiricisi, kök hücrelerin diferansiyasyon yeteneği üzerinde yaygın olarak etkilidir ve bu konuda seçici olmayan bir rol oynamaktadır. Üç farklı dozda 5-azasitidine maruz kaldıktan sonra, kemik iliği mezenkimal kök hücrelerinin kardiyomiyosit ve miyosit benzeri hücrelere diferansiyasyon yeteneği irdelendi ve karşılaştırıldı. Bu çalışmanın amacı, 5-azasitidinin insan mezenkimal kök hücrelerinin (MKH) kardiyomiyosit ve miyosite diferansiyasyonunu sağlamak için gerekli olan etkin dozunun tespit edilmesidir.

**Gereç ve Yöntemler:** İnsan kemik iliği aspirasyonları sağlıklı donörlerden yapıldı. MSCler osteoblast ve adipozit farklılaşması için kültüre edildi. Osteojenik veya adipojenik özellikler immunositokimyasal boyama ile incelendi. BMMSCler tripsinize edilerek tek hücre süspansiyonu elde edildi ve akım sitometri için hazırlandı. MSClere 5, 10 veya 15 μM 5-azacytidine 24 saat uygulandı, daha sonra 3 hafta kültüre edildi. Total RNA 5-azacytidine uygulanan ve uygulanmayan hücrelerden elde edildi. Troponin T ve GATA4 antikorları kardiyojenik belirteçler, myojenin ve MyoD antikorları myosit belirteçleri olarak kullanıldı.

**Bulgular:** 5-Azasitidinin her 3 dozuna da maruz kalmış MKH’lerin morfoloji ve büyüme hızları, kontrol MKH’lerin morfoloji ve büyüme hızları ile benzerdi. İmmünfloresans yöntemi ile kalbe özgül belirteçler olan GATA4 ve troponin T ile çizgili kasa özgül belirteçler olan MyoD ve miyojenin sunumlarının incelenmesi sonucunda, 15 μM 5-azasitidine maruz kalan hücrelerde bu belirteçlerin kuvvetli pozitif olduğu görüldü. Gerçek zamanlı polimeraz zincir reaksiyonu sonuçları incelendi; 15 μM 5-azasitidine maruz kalan MKH’lerde, daha düşük doz 5-azasitidin alan veya 5-azasitidin uygulanmayan MKH’lere göre kalp ve çizgili kasa özgül mRNA’ların daha yüksek oranda sunum düzeyleri olduğu tespit edildi.

**Sonuç:** 5-Azasitidine maruz bırakılan MKH’lerin kardiyomiyosit ve miyositlere diferansiye olma yeteneği olduğu ve en uygun dozun 15 μM 5-azasitidin olabileceği gösterildi. MKH’lerin özgül hücre tiplerine diferansiyasyonunu incelemek için bunu etkileyebilecek diğer faktörlerin de irdelenmesi gerekmektedir.

## INTRODUCTION

Mesenchymal stem cells (MSCs) provide a promising approach for cellular therapy because of their self-renewing properties and multipotent differentiation capacity. MSCs were discovered by Friedenstein from bone marrow cultures and reported to be marrow stromal cells [1]. Since 1993, Pittenger and colleagues [2] have explored the characteristics and multipotent capabilities of these human bone marrow-derived cells. MSCs possess the following traits: plastic adherent properties; positivity for the cell surface markers CD105, CD90, and CD44; and an ability to differentiate into a mesodermal lineage (osteogenic, adipogenic, and chondrogenic cells) [3]. At present, MSCs from other sources have been found; these MSCs demonstrate efficiencies that are similar to the efficiencies of bone marrow-derived MSCs (BMMSCs) [4,5]. MSCs are very intriguing cells for therapeutic purposes because of both their capacity to differentiate into cells of a mesodermal lineage and their immunoregulatory roles [6,7].

The ability of MSCs to differentiate into specialized cells has been well studied; in particular, the differentiation of MSCs into cardiomyocytes and myocytes has been extensively investigated [8] because stem cell-derived cardiomyocytes could prove extremely useful for addressing the myocardiogenic suffering of many patients [9]. Most of these in vitro studies have examined MSCs that have been treated with specific growth factors [10] and histone modifiers [11]. Through its function as a DNA methyltransferase inhibitor, 5-azacytidine can affect histone and DNA methylation and cause DNA hypomethylation. However, high doses of 5-azacytidine produce cytotoxic effects. Therefore, a low dose of 5-azacytidine was selected for use as a differentiation inducer in stem cells [12]. Given that DNA methylation can govern gene silencing activity [13], the hypomethylation activity of 5-azacytidine may play a nonspecific role in upregulating the differentiation-promoting factors of stem cells. However, skeletal and cardiac muscle differentiation from human stem cells through the use of 5-azacytidine remains controversial; moreover, 5-azacytidine has not yet been proven to produce completely differentiated cells. To investigate the effect of 5-azacytidine on the cardiomyogenic differentiation of BMMSCs, bone marrow mesenchymal stem cells were treated with low doses of 5, 10, or 15 μM 5-azacytidine, and the resulting expression levels of skeletal- and cardiac-specific genes were examined.

## MATERIALS AND METHODS

**Sample Preparation**

Human bone marrow aspirations were collected from healthy donors who had completed written consent forms that were approved by the Siriraj Institution Review Board (SIRB) under permission No.si587/2008, which was approved on 30 October 2012 to be revised every 1 year. Each heparinized bone marrow aspiration sample was diluted with phosphate buffered saline (PBS) before being layered onto an equal volume of Ficoll-Hypaque density gradient. The sample was centrifuged at a speed of 2000 rpm for 30 min at room temperature. The interphase layer of mononuclear cells was transferred into new centrifugal tubes and then washed twice. The mononuclear cells were suspended in Dulbecco’s modified Eagle’s medium (DMEM) supplemented with 10% fetal bovine serum (FBS), 100 U/mL penicillin, and 100 μg/mL streptomycin (complete medium), and the cells were seeded at 1x106 cells per T25 culture flask. The cells were cultivated in 5% CO2 at 37 °C for 5-7 days; the nonadherent cells were then removed, whereas the plastic-adherent cells (the MSCs) were maintained under the same culture conditions. The MSCs were maintained and were subcultured with 0.25% trypsin with EDTA; cells from passages 3-5 were employed for this study.

**The Osteogenic and Adipogenic Differentiation of MSCs**

MSCs at passage numbers 3-5 were used for the study of mesodermal differentiation. Cells were cultured in 6-well plates at seeding densities of 1x10^4^ cells/mL in lineage- specific differentiation media. MSCs were cultured in NH OsteoDiff medium (Miltenyi Biotec, Germany) to promote osteoblast differentiation and in NH AdipoDiff medium (Miltenyi Biotec) to promote adipocyte differentiation; in both cases, the medium was changed twice weekly. These culture conditions were maintained for 2-3 weeks, and the evaluation of osteogenic or adipogenic properties was then performed through immunocytochemical staining with alkaline phosphatase or Oil Red O (Sigma-Aldrich, USA), respectively, as well as through morphological observations.

**Flow Cytometry**

BMMSCs from bone marrow at passages 3-5 were trypsinized with 0.5% trypsin-EDTA into single-cell suspensions and then washed twice with PBS. Cell suspensions containing 5x10^5^ cells in a total volume of 50 μL were labeled with the following mouse antihuman antibodies, which were conjugated with either fluorescein isothiocyanate (FITC) or phycoerythrin (PE): CD34-PE (BD Pharmingen, USA), CD45-FITC (BD Pharmingen), CD73-PE (BD Pharmingen), CD90-FITC (AbD SeroTec, USA), and CD105-FITC (AbD SeroTec). The cells were incubated with the appropriate antibodies at 4 °C for 30 min in the dark. The labeled cells were washed twice with cold PBS and centrifuged at 2000 rpm for 5 minutes at 4 °C; the cells were then fixed with 1% paraformaldehyde. The samples were assessed with a flow cytometer (FACS CaliburTM, Becton Dickinson, USA), and the flow cytometry results were analyzed with the CellQuest software package (Becton Dickinson).

**MSCs and 5-Azacytidine Treatments**

MSCs of passages 3-5 were seeded at a density of 1x10^5^ cells/ mL and cultured in a basal medium of DMEM supplemented with 10% FBS and penicillin-streptomycin. Three different doses of 5-azacytidine (Sigma-Aldrich), namely 5 μm, 10 μm, and 15 μm, were applied to MSC samples for 24 h before the culture medium was changed to the complete medium. The culture conditions were maintained continuously for 2-3 weeks to observe the morphology, growth, and percent viability of the MSCs; MSCs from these cultures were then assessed through real-time RT-PCR and immunofluorescence studies for cardiomyocytes and myocytes.

**Quantitative Real-Time PCR**

Total RNA was extracted from untreated and 5-azacytidine– treated cells after 1-3 weeks of culture using TRIzol® (Invitrogen, USA) in accordance with the manufacturer’s protocol. The RNA sample was diluted in DEPC water before it was quantified by spectrophotometry at 260 nm. The 2-μg RNA sample was reverse-transcribed into cDNA with Superscript III First-Strand Synthesis (Invitrogen) following the manufacturer’s instructions. Quantitative real-time PCR (qRT-PCR) analyses for cardiac- and myogenic-specific genes were performed with the following primers: α-cardiac actin (260 bp), sense 5’-TCTATGAGGGCTACGCTTTG-3’ and antisense 5’-GCCAATAGTGATGACTTGGC-3’; troponin T (225 bp), sense 5’-AGAGCGGAAAAGTGGGAAGA-3’ and antisense 5’-CTGGTTATCGTTGATCCTGT-3’; myogenin, sense 5’-GCCAGACTATCCCCTTCCTC-3’ and antisense 5’-GAGGCCGCGTTATGATAAAA-3’; Myf5 (167 bp), sense 5’-AATTTGGGGACGAGTTTGTG-3’ and antisense 5’-CATGGTGGTGGACTTCCTCT-3’; and GAPDH (139 bp), sense 5’-GTCAACGGATTTGGTCGTATTG-3’ and antisense 5’-CATGGGTGGAATCATATTGGAA-3’.

The PCR amplifications, which used SYBR® Green PCR (Applied Biosystems, USA), were run for 40 cycles, each of which involved the following steps: a denaturation step for 10 s at 95 °C, an annealing step for 10 s at 60 °C, and an extension step for 40 s at 72 °C. The expression levels of cardiogenic- and myogenic-specific markers from 5-azacytidine–treated cells and untreated cells were normalized with glyceraldehyde-3-phosphate dehydrogenase (GAPDH). The gene expression levels of untreated cells and treated cells were then investigated with the comparative CT method. The observations were performed with an ABI 7500 Fast Real-Time PCR System (Applied Biosystems), and the resulting data were analyzed with the ABI 7500 software package, version 2.0.3 (Applied Biosystems).

**Immunofluorescence Studies**

MSCs were cultured in 4-well chamber slides (Nunc, USA) in complete medium for 24 h under one of the following conditions: no 5-azacytidine, 5 μM 5-azacytidine, 10 μM 5-azacytidine, or 15 μM 5-azacytidine. After this period, the medium was changed to complete growth medium and maintained for 7 days. Troponin T (Santa Cruz Biotechnology, Inc., USA) and GATA4 antibodies (Millipore, USA) were used as cardiogenic markers, whereas myogenin (Millipore) and MyoD antibodies (Millipore) were used as myocyte markers. The growth medium was completely removed before the cells were washed twice with PBS. The cells were then fixed in 4% paraformaldehyde in PBS for 30 min. Following this fixation, the fixative was removed and nonspecific binding blocking using 1% bovine serum albumin was performed. The appropriate primary antibody was added for 45 min at room temperature and washed twice with PBS. The FITC- or rhodamine-conjugated secondary antibody was added to cover the cell layer for 45 min in the dark. The cells were then washed twice with PBS solution and counterstained with DAPI before they were observed under a fluorescent microscope. 

**Statistical Analysis**

The data analysis was performed with SPSS 11.0 (SPSS Inc., USA). The expressions of cardiogenic and myogenic genes in the MSCs that had been treated with 3 different doses of 5-azacytidine were compared with the expressions of those genes in untreated cells. The gene expressions are presented in terms of means ± standard errors, and the Mann- Whitney U test was used for significance assessments; in this study, P<0.05 was considered to be statistically significant.

## RESULTS

**The Cultures and Characterization of Human BMMSCs**

**Figure 1:**
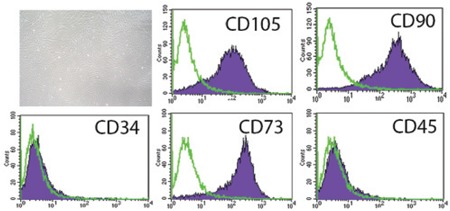
In culture, MSCs presented a spindle-shaped and fibroblast-like morphology. Various cell surface markers were analyzed by flow cytometry; positive results were obtained for CD73, CD90, and CD105 (MSC markers), whereas negative results were obtained for CD34 and CD45 (hematopoietic markers).

**Figure 2:**
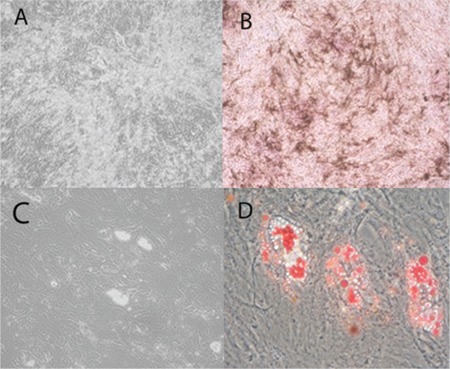
The characterization of BMMSCs that have been induced to differentiate into osteoblast-like cells (A) or adipocyte-like cells (C). The cells were analyzed by cytochemical staining, and BMMSCs that were cultured in osteogenic conditions were positive for phosphatase (B), whereas BMMSCs that were cultured in adipogenic conditions were positive for Oil Red O (D).

Bone marrow-derived MSCs were isolated from aspirated bone marrow based on their property of plastic adherence. The MSCs were grown until they reached confluence; cells that had been passaged between 3 and 5 times were used for this study. The MSCs in culture demonstrated spindle- shaped and fibroblast-like morphology (Figure 1). The MSCs were then characterized in terms of their cell surface markers and mesodermal differentiation capacity. The MSC surfaces were positive for CD73, CD90, and CD105 (MSC markers) but negative for CD34 and CD45 (hematopoietic markers) (Figure 1). The properties of MSCs that had differentiated into distinct lineages were evaluated for morphological changes and cytochemical staining. The morphological changes of MSCs that were cultured in osteogenic medium or adipogenic medium were observed (Figures 2A and 2C). The cytochemical staining was then performed and indicated that MSCs that were cultured in osteogenic medium were positive for alkaline phosphatase (Figure 2B) and that MSCs that were cultured in adipogenic medium were positive in Oil Red O stains (Figure 2D).

**5-Azacytidine–treated BMMSCs**

**Figure 3:**
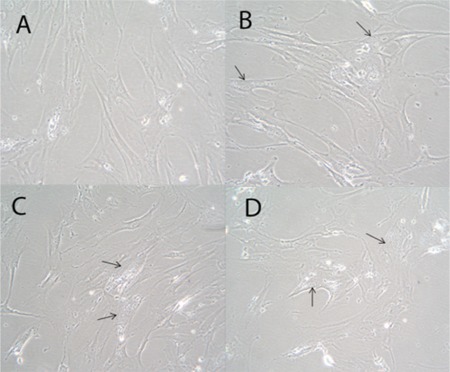
BMMSC morphology was spindle-shaped in the growth medium for both untreated cells (A) and cells that had been treated with 5-azacytidine (B, C, D). The cells in the treated group were broader and displayed increased granular content around their nucleus (arrow) (B, C, D).

**Figure 4:**
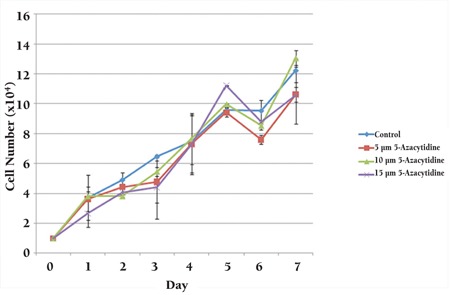
A comparison of the growth curves of untreated BMMSCs (control) and BMMSCs that had been treated with 1 of 3 different concentrations (5, 10, or 15 μM) of 5-azacytidine for 24 h. The results were similar in both the untreated (control) and treated groups.

MSCs were cultured in growth medium and treated with 1 of 3 different concentrations of 5-azacytidine (5, 10, or 15 μM) for 24 h. Subsequently, after 3 additional weeks of culture, the treated cells were compared with untreated cells. The morphological changes over time were observed; these did not differ for various doses of the 5-azacytidine. Both treated and untreated MSCs displayed spindle-shaped appearances. Compared with the untreated cells (Figure 3A), the treated cells were broader and possessed increased granular content around their nuclei (Figures 3B-3D). As shown in Figure 4, the growth of MSCs that were treated with any of the 3 different concentrations of 5-azacytidine for 24 h was similar to the growth of the control group. 

**Immunofluorescence**

**Figure 5:**
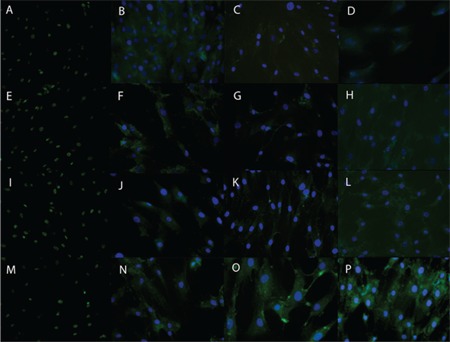
The immunofluorescence of BMMSCs that were either left untreated (control) or treated with 1 of 3 different concentrations of 5-azacytidine for 24 h: 0 (A-D), 5 (E-H), 10 (I-J), or 15 (M-P) μM. The BMMSCs were first immunostained with antibodies for cardiac-specific markers (green) GATA4 (A, E, I, M) and troponin T (B, F, J, N) and for skeletal-specific markers (green) MyoD (C, G, K, O) and myogenin (D, H, L, P), then incubated with an appropriate FITC- or rhodamine-conjugated secondary antibody for 45 min, counterstained with DAPI (blue), and finally visualized.

Untreated BMMSCs and BMMSCs that had been treated for 24 h with 1 of 3 concentrations of 5-azacytidine (5, 10, or 15 μM) were maintained in complete media for 7 days. Immunostaining was performed to study the expression of cardiac-specific proteins (GATA4 and troponin T) and skeletal-specific proteins (MyoD and myogenin). The expression of the examined cardiac- and skeletal-specific markers was observed in both treated and untreated BMMSCs. The highest signal intensity from these markers was displayed by the cells that had been treated with 15 μM 5-azacytidine (Figure 5).

**Quantitative Real-time PCR**

**Figure 6:**
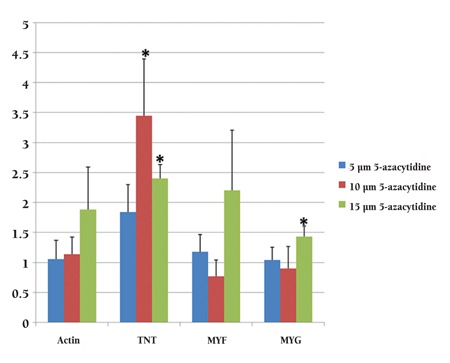
qRT-PCR demonstrated that compared with the expression levels of control cells, the expression level for myogenin was significantly higher in the cells that had been treated with 15 μM 5-azacytidine, and troponin T expression levels were significantly higher in the cells that had been treated with either 10 μM or 15 μM 5-azacytidine (p <0.05).

To validate the observed upregulation of cardiac- and skeletal muscle-specific proteins, the levels of mRNAs that encode several cardiac- and skeletal muscle-specific genes were determined by qRT-PCR. The PCR results indicated that after 7 days, compared with untreated cells, 5-azacytidine– treated cells demonstrated increased expression levels of various cardiac- and skeletal muscle-specific genes, including α-cardiac actin, troponin T, Myf5, and myogenin. Four genes were expressed in both treated and untreated BMMSCs (data not shown). However, compared with the expression levels that were observed in untreated control cells, the myogenin expression level was significantly higher in the cells that had been treated with 15 μM 5-azacytidine, and the troponin T expression level was significantly higher in the cells that had been treated with either 10 or 15 μM 5-azacytidine (Figure 6; p <0.05). The expressions of both cardiac-specific genes (α-cardiac actin, troponin T) and skeletal muscle-specific marker genes (MyoD, myogenin) were increased only during the first week and subsequently decreased in the later weeks (data not shown).

## DISCUSSION

Stem cells represent promising sources of future clinical applications. Because stem cells can not only self-renew but also differentiate into specialized cells, the potential of stem cells to support cell therapies has been widely studied, particularly with respect to the differentiation properties of these cells. The treatment of stem cells with the DNA methylating agent 5-azacytidine has been demonstrated to induce the differentiation of stem cells into multiple cellular phenotypes, including cardiomyocytes [14,15]; however, the mechanism underlying the effects of 5-azacytidine has not yet been elucidated. Konieczny and Emerson [16] suggested that 5–azacytidine can hypomethylate a myogenic- determinant locus, resulting in the transcriptional activation of this locus and thereby allowing for the induction of myogenic differentiation. Because of its hypomethylation effects, 5-azacytidine has also been shown to induce nonspecific expression on a variety of genes [17].

Human BMMSCs are a type of multipotent stem cell that possess the potential to differentiate into many cell types that are derived from the mesoderm, such as osteoblasts [18], myocytes [19], and cardiomyocytes [20]. To investigate the effects of 5-azacytidine on the cardiogenic and myogenic differentiation of BMMSCs, bone marrow mesenchymal stem cells were treated with low doses of 5-azacytidine (5, 10, or 15 μM), and the expression of skeletal muscle- and cardiac-specific genes was compared. We cultured MSCs with the 5-azacytidine for 24 h before allowing the cells to grow in complete medium for 3 weeks. The expressions of both cardiac-specific genes (α-cardiac actin, troponin T) and skeletal muscle-specific marker gene (MyoD, myogenin) were increased in the examined cells, but only during the first week; these expression levels decreased in subsequent weeks. This result suggests that 5-azacytidine can enhance the expression of the examined genes for a short period of time [17]. Thus, to promote differentiation of MSCs into cardiomyocyte-like cells, the administration of additional exogenous growth factors or cytokines as co-inducers in combination with 5-azacytidine may be required [10]. A few potential inducers that were previously investigated are IGF-1, oxytocin, bFGF, cardiotrophin, and TGF-β1 [21,22,23].

From our study, it appears that the treatment of MSCs with 5-azacytidine can promote the expression of both cardiac-specific genes (α-cardiac actin, troponin T) and skeletal muscle-specific marker genes (MyoD, myogenin). We also studied the effect of 5-azacytidine on the percent viabilities of cells for 1 week, but no change in viability was observed for the 3 5-azacytidine concentrations that were tested. Moreover, the 15 μM 5-azacytidine–treated cells displayed higher myogenin expression than control cells. Troponin T also demonstrated its highest expression levels in cells that had been treated with 10 μM or 15 μM 5-azacytidine; these results are consistent with the findings of previously published studies on the differentiation of MSCs into cardiomyocyte-like cells [24,25].

In conclusion, our study demonstrated that 15 μM 5-azacytidine can promote the expression of cardiac- and skeletal muscle-specific genes in BMMSCs in culture. The in vitro treatment of BMMSCs with 5-azacytidine supports initial cardiomyogenic differentiation over a relatively short period of time (approximately 1 week). Thus, 5-azacytidine might be used as a pretreatment to induce BMMSCs towards complete cardiac differentiation. However, factors that promote specific cell types have not yet been elucidated, and this topic still merits further study.

**Acknowledgments:** This study was supported by the Thailand Research Fund (Grant no. MRG5080187) and the Commission on Higher Education (Grant no. CHERES- RG-49). S. Issaragrisil is a Senior Research Scholar of the Thailand Research Fund.
